# Factors Associated with Family Caregivers’ Intentions to Complete an Advance Directive for Individuals with Dementia: A Cross-Sectional Descriptive Study

**DOI:** 10.3390/healthcare13111297

**Published:** 2025-05-29

**Authors:** Hyeseon Park, Sujin Kang, Youngji Kim

**Affiliations:** 1 Intensive Care Unit, Yuseong Sun Hospital, Daejeon 34084, Republic of Korea; 2 Department of Nursing, The Catholic University of Korea, Seoul 06591, Republic of Korea; 3 Department of Nursing, Kongju National University, Gongju-si 32588, Republic of Korea

**Keywords:** advance directives, caregivers, dementia, terminal care

## Abstract

**Background/Objectives:** As dementia progresses, patients often lose decision-making capacity, leaving family members responsible for making critical end-of-life (EOL) care decisions. This cross-sectional descriptive study explored the factors associated with the intention to complete an advance directive (AD) among family caregivers of older adults with dementia in South Korea. **Methods**: This study surveyed 140 caregivers aged 20 years or older to assess their knowledge of advance directives (ADs), preferences for EOL care, and attitudes toward withdrawing life-sustaining treatment (LST). Data were collected from 1 August to 14 August 2024, and analyzed using descriptive statistics, the Chi-squared test, Fisher’s exact test, Spearman’s correlation, and logistic regression, with SPSS/WIN 28.0. **Results**: The intention to complete an AD significantly differed by religion (*p* = 0.004) and the functional status of the patient with dementia (*p* = 0.012). There was a positive correlation between intention to complete an AD and knowledge of ADs (r = 0.23, *p* = 0.007). Factors associated with intention to complete an AD included religion (OR = 4.36, *p* = 0.028) and knowledge of ADs (OR = 1.16, *p* = 0.033), explaining 22.2% of the variance, which is considered meaningful in studies dealing with complex psychosocial and behavioral variables. **Conclusions**: These findings suggest that religious affiliation and knowledge of ADs may contribute to greater emphasis on EOL preparedness and self-determination. To promote informed decision making, it is essential to implement targeted educational interventions—such as community-based workshops, telehealth counseling, and in-clinic guidance—that enhance caregivers’ understanding of ADs and empower them in their caregiving roles.

## 1. Introduction

As of 2021, approximately 57 million people worldwide were living with dementia, with over 60% residing in low- and middle-income countries. Nearly 10 million new cases are diagnosed globally each year [1]. The prevalence of dementia in South Korea continues to increase as the population ages [2]. Dementia is an irreversible degenerative disease that not only leads to early cognitive decline but also causes neurological impairments, incontinence, infections, and other physical complications [3]. Therefore, it is considered a condition that requires appropriate end-of-life (EOL) care and advance care planning (ACP) [1]. Due to the prolonged caregiving period, patients may feel that they are a burden to their families, highlighting the need for discussions on ACP and advance directives (ADs) for patients with dementia and their family caregivers [4]. However, predicting the progression and deterioration of dementia is challenging, making it difficult to determine the appropriate timing for discussions regarding ADs and ACP with patients [5,6].

An “advance directive (AD)” refers to a document in which an individual aged 19 or older personally records their wishes regarding decisions on life-sustaining treatment (LST) and hospice care [7]. However, under the current law, patients with dementia alone are not eligible for hospice care, and there are no available data on the actual proportion of patients with dementia who have completed an AD. In cases where patients with dementia experience severe cognitive impairment, they become unable to express their intentions regarding ADs, leaving decisions related to LST entirely up to the opinions of their family caregivers [8].

However, the level of agreement between patients with dementia and their family caregivers regarding ACP is low, and research on family caregivers’ awareness of ADs for patients with dementia remains significantly insufficient [9]. While a large portion of the general public has basic knowledge about ADs, they lack the understanding of legal aspects and detailed implementation procedures [10,11]. Therefore, the necessity for more proactive promotion and the provision of accurate information regarding ADs has been emphasized.

Preferences for EOL care refer to the wishes of terminally ill patients regarding the physical, emotional, technical, spiritual, and decisive aspects of their care due to illness, injury, or disease [12]. Previous studies have shown that most individuals prefer pain management and other measures to alleviate suffering rather than LST aimed at prolonging life meaninglessly [13,14,15]. However, many patients undergo unnecessary and burdensome treatments due to difficulties in completing ADs at the appropriate time, a lack of experience among healthcare professionals, and disagreements between family caregivers and patients [16].

Withdrawal of LST refers to the discontinuation of life-prolonging medical interventions, allowing terminally ill patients to face death with dignity by avoiding unnecessary and excessive treatment [17]. In the case of patients with dementia, most decisions regarding EOL care are made by their family caregivers. However, caregivers often take a passive stance toward ACP due to concerns about the potential negative impact on the patient. This indicates that family caregivers of elderly patients with dementia experience significant psychological burden and stress [18].

Considering these factors, preparing and understanding ADs in advance can not only alleviate the psychological burden on family caregivers but also support them in fulfilling their appropriate role while respecting the patient’s rights and dignity when facing ethical dilemmas. Therefore, it is necessary to examine family caregivers’ awareness and knowledge of ADs, and their preferences for EOL care. However, research focusing on family caregivers remains scarce.

Thus, this study was conducted to examine how family caregivers’ knowledge of ADs, preferences for EOL care, and attitudes toward withdrawing LST influence their intention to complete an AD. The findings aim to provide foundational data for developing educational and institutional interventions that enhance awareness and support informed decision making, thereby promoting patient dignity and appropriate EOL care.

## 2. Materials and Methods

### 2.1. Study Design

This study is a cross-sectional descriptive survey designed to examine the effects of family caregivers’ knowledge of ADs, preferences for EOL care, and attitudes toward withdrawing LST on their intention to complete an AD.

### 2.2. Participants

This study targeted 140 family caregivers of elderly patients with dementia nationwide who voluntarily agreed to participate after understanding this study’s purpose. The inclusion criteria required participants to be adult family caregivers aged 20 or older who were caring for a patient with dementia aged 65 or older. The patient with dementia had to be receiving either inpatient or outpatient treatment and required assistance in daily activities. This functional status meant that the patient needed verbal instructions or physical assistance for tasks such as eating and other daily activities.

Exclusion criteria included individuals who were licensed healthcare professionals currently practicing in clinical settings (such as physicians and nurses, as defined by medical law), individuals diagnosed with mental illness or taking related medications, and family caregivers whose patient with dementia had an independent functional status, meaning they could perform most daily activities without special instructions or assistance [19].

The sample size was determined using the G*Power 3.1.9.7. (Düsseldorf University, Düsseldorf, Germany). Since no prior studies had examined family caregivers’ intention to complete an advance directive, related studies conducted with other populations were referenced [20,21]. In a logistic regression analysis with OR = 2.0, α = 0.05, and power = 0.80, the required sample size was calculated to be 113 participants. Considering a 20% dropout rate, a total of 140 participants were recruited, and there were no dropouts.

### 2.3. Data Collection

From 1 August to 14 August 2024, participants were recruited by contacting administrators of online communities for family caregivers of patients with dementia nationwide. After explaining the purpose and significance of this study, permission was obtained to post a survey advertisement, and an online survey was subsequently conducted.

Before the survey began, participants were provided with a detailed explanation of this study’s purpose. It was emphasized that participation was voluntary, all survey responses would remain anonymous, and the data would be used solely for research purposes. Participants were also informed that they could withdraw from the survey at any time. The survey was conducted via an online survey form, and participants provided informed consent before proceeding. The survey took approximately 15 min to complete.

### 2.4. Measurements

#### 2.4.1. General Characteristics of Participants

The characteristics of the participants were assessed using a structured questionnaire developed based on prior studies [19,21]. The questionnaire included both multiple-choice and open-ended self-report items, which participants completed during an online survey. It comprised 11 items: age, gender, religion, education level, income level, type of family cohabitation, presence of other family members assisting with dementia care, subjective health status, relationship to the patient with dementia, functional status of the patient with dementia, and type of dementia care provided.

#### 2.4.2. Knowledge of ADs

Knowledge of ADs was measured using the end-of-life care decision inventory (EOL-CDI), a tool originally developed by Hong & Kim (2013) [22] and later revised and supplemented by Kim et al. (2021) [23] following amendments to relevant laws. This tool consists of 21 items, with response options of “Yes”, “No”, and “I don’t know.” Incorrect answers and “I don’t know” responses were scored as 0 points, while correct answers were scored as 1 point. The total score ranged from 0 to 21, with higher scores indicating a greater level of knowledge about advance directives. The reliability of the tool at the time of development was Cronbach’s α = 0.81, and, in this study, the reliability was Cronbach’s α = 0.66.

#### 2.4.3. Preference for EOL Care

The Preferences for Care near the End of Life (PCEOL) scale, developed by Gauthier and Froman (2001) [12], was used in this study. The Korean version of this tool, revised and supplemented by Lee and Kim (2009) [24], was utilized for measurement. This tool consists of a total of 26 items, categorized into the following subdomains: autonomous decision making (8 items), spirituality (6 items), role of family (5 items), decision making by healthcare professionals (4 items), pain management (3 items). Each item is rated on a 5-point Likert scale ranging from 1 (strongly disagree) to 5 (strongly agree). A higher score indicates a clearer preference for EOL care. The reliability of the tool was reported as Cronbach’s α = 0.68–0.97 in the study by Gauthier and Froman (2001) [12] and Cronbach’s α = 0.42–0.89 in the study by Lee and Kim (2009) [24]. In this study, the overall reliability was Cronbach’s α = 0.84, with subdomain reliability ranging from Cronbach’s α = 0.41–0.90.

#### 2.4.4. Attitudes Toward Withdrawing LST

Attitudes toward withdrawing LST were measured using a tool originally developed by Park (2000) [25] and later revised and supplemented by Byun et al. (2003) [26]. This tool consists of 19 items, with 15 items assessing a positive attitude toward withdrawing LST and 4 items measuring a negative attitude toward it. Each item is rated on a 5-point Likert scale ranging from 1 (strongly disagree) to 5 (strongly agree). A higher score indicates a stronger agreement with the withdrawal of LST. The reliability of the tool was reported as Cronbach’s α = 0.88 in the study by Byun et al. (2003) [26], and, in this study, the reliability was Cronbach’s α = 0.77.

#### 2.4.5. Intention to Complete an AD

The intention to complete an advance directive was measured with a single question: “Do you intend to complete an advance directive?” Responses were coded as “Yes” (1 point) or “No/Undecided” (0 point) [21].

### 2.5. Data Analysis

The collected data were analyzed using SPSS WIN 28.0 program (IBM Corp., Armonk, NY, USA). Descriptive statistics, including frequency, percentage, mean, and standard deviation, were used to examine participants’ general characteristics and their intention to complete an advance directive. The levels of knowledge about ADs, preference for EOL care, and attitudes toward the withdrawal of LST were analyzed using means and standard deviations. Differences in the intention to complete an AD based on participants’ general characteristics were analyzed using the Chi-square test and Fisher’s exact test. The relationships between knowledge of ADs, preference for EOL care, attitudes toward the withdrawal of LST, and the intention to complete an AD were examined using Spearman’s correlation coefficient. Factors associated with the intention to complete an AD were analyzed using logistic regression.

## 3. Results

### 3.1. Study Population

Among the total of 140 participants, 120 (85.7%) were female. In terms of age, 62 (44.3%) were in their 40s, and 46 (32.9%) were in their 30s, with an average age of 41.5 ± 8.48 years. Regarding religion, 61 (43.6%) had a religion, while 79 (56.4%) did not. The income level was “average” for 101 participants (72.1%), and the highest educational attainment was a bachelor’s degree (university graduate) for 104 participants (74.3%). The type of cohabitation with family members showed that 85 participants (60.7%) were living with an older adult with dementia. Among those caring for a patient with dementia, 107 participants (76.4%) had other family members helping with care. The subjective health status indicated that 80 participants (57.1%) considered themselves to be healthy. The relationship with an older adult with dementia was most commonly “spouse or child” for 103 participants (73.5%). The functional status of an older adult with dementia required “moderate or greater levels of assistance” for 90 participants (64.3%). The type of dementia care provided was “receiving full-time care from family members” for 61 participants (43.6%), which was the largest proportion (Table 1).

### 3.2. Knowledge of ADs, Preferences for EOL Care, Attitudes Toward Withdrawing LST, and Intention to Complete an AD

The mean score for knowledge of ADs was 12.65 ± 3.30 out of 21, with a mean percentage of correct responses (60.7%) (Appendix A). The mean score for preferences for EOL care was 3.12 ± 0.56 out of 5 points. Among the subdomains, the highest mean score was for pain management items at 3.96 ± 0.68, followed by role of family items at 3.64 ± 0.97, spirituality items at 3.44 ± 1.04, decision making by healthcare professional items at 3.07 ± 1.13, and autonomous decision making at 2.28 ± 0.83. The attitudes toward withdrawing LST had a mean score of 3.90 ± 0.50 out of 5 points. Regarding the intention to complete an AD, 119 participants (85%) indicated that they had the intention to complete one, while 21 participants (15%) reported that they did not (Table 2).

### 3.3. Differences in the Intention to Complete an AD According to General Characteristics

The intention to complete an advance directive showed significant differences based on religion (*p* = 0.004) and the functional level of the patient with dementia (*p* = 0.012). Participants without a religious affiliation had an intention rate of 77.2%, while those with a religious affiliation had a higher intention rate of 95.1%. Regarding the functional level of the patient with dementia, those requiring simple verbal instructions or minimal assistance had an intention rate of 74.0%, whereas those requiring moderate or greater levels of assistance had a higher intention rate of 91.1%. Meanwhile, the intention to complete an AD did not show significant differences according to age, gender, education level, income level, family cohabitation type, presence of other family members providing care, subjective health status, relationship to the patient, or type of dementia care provided (Table 3).

### 3.4. Correlation Between Knowledge of ADs, Preferences for EOL Care, Attitudes Toward Withdrawing LST, and Intention to Complete an AD

Among family caregivers of patients with dementia, the intention to complete an advance directive showed a significant positive correlation with knowledge of ADs (r = 0.23, *p* = 0.007). On the other hand, the intention to complete an AD did not show a significant correlation with preferences for EOL care or attitudes toward withdrawing LST (Table 4).

### 3.5. Factors Associated with the Intention to Complete an Advance Directive

To identify the factors influencing participants’ intention to complete an AD, a logistic regression analysis was conducted. The analysis included gender, religion, functional level of the patient with dementia, and knowledge of ADs, as these variables showed significant differences to or correlations with the intention to complete an AD.

The results of the logistic regression analysis indicated that religion (OR = 4.36, *p* = 0.028) and knowledge of ADs (OR = 1.16, *p* = 0.033) were significant influencing factors. Specifically, individuals with a religion were 4.36 times more likely to express an intention to complete an AD compared with those without a religion. Additionally, a higher score in knowledge of ADs was associated with a 1.16 times greater likelihood of intending to complete an AD. The explanatory power of the study model, represented by Nagelkerke R² = 0.222, indicated that the included variables explained 22.2% of the variance in the intention to complete an advance directive (Table 5).

## 4. Discussion

This study aimed to assess the level of knowledge about ADs, the intention to complete an AD, and the associated factors among family caregivers of patients with dementia—whose right to self-determination may be compromised—in order to support the effective planning of EOL care for patients with dementia.

This study found that the average knowledge score of caregivers regarding ADs was 12.65 out of 21 points. This score is comparable to that reported in a previous study involving caregivers of individuals with dementia, which utilized the Alzheimer’s Disease Knowledge Scale [27]. However, it was slightly lower than the score (14.59 out of 21 points) observed in a separate study [23] assessing clinical nurses using the same instrument. These findings suggest that, unlike healthcare professionals, family caregivers may have limited access to information regarding ADs, likely due to their non-medical background.

The overall mean score for preferences for EOL care was 3.12 out of 5. Among the subdomains, pain management had the highest score (3.96), followed by the role of family (3.64), spirituality (3.44), decision making by healthcare professionals (3.07), and autonomous decision making (2.28). This indicates that participants consider pain relief the most important aspect of EOL care and tend to trust the decisions of family members or healthcare professionals rather than their own autonomous decision making. These findings are consistent with the study by Jung and Park (2024) [28]. The prioritization of family or professional decisions over individual preferences may reflect collectivist cultural values, highlighting the need for further research on how cultural norms uniquely influence caregiver decision making. In Korean society, the tradition of filial piety (hyo) and family-centered decision making remains strong, meaning that family opinions and roles significantly influence EOL treatment and care decisions, alongside patient autonomy [29]. Particularly for patients with dementia with diminished decision making capacity, family caregivers’ opinions are prioritized, which may influence both the intention to complete an AD and actual completion. Therefore, this cultural specificity must be carefully considered in the design of future policies and educational programs.

Due to the unpredictable progression of dementia, it is essential for caregivers to recognize and honor the patient’s preferences regarding EOL care to uphold their dignity [5,6]. However, because patients with dementia are often perceived as having lost their decision-making capacity, families who must make decisions on their behalf are known to experience a high level of burden [30]. Particularly, in the case of patients with dementia where autonomous decision making is often challenging due to the nature of the disease, the role of family caregivers becomes even more crucial. Therefore, it is essential to provide education and intervention programs that support family caregivers in recognizing the EOL care preferences of patients with dementia and effectively incorporating them into ACP.

Attitudes toward withdrawing LST were measured at an average score of 3.90, which was higher than those reported in previous studies conducted on older adults [31]. Notably, the statement “Patients have the right to decide their own death” received a high score (Appendix B), indicating a positive attitude toward discontinuing medically futile treatment for terminally ill patients. However, there was still a demand for basic medical interventions, such as fluid therapy and antibiotics, and attitudes toward withdrawing LST for organ donation were relatively less favorable (Appendix B). These results suggest that many people are not familiar with information related to organ transplantation and that various factors, such as fear of losing bodily integrity or religious beliefs, may have contributed to negative perceptions of organ transplantation [32]. Considering these aspects, policy improvements are needed to support thorough discussions between patients and caregivers during the decision-making process for LST.

The intention to complete an AD was observed in 85% of the participants, which was higher than in previous studies [21]. This suggests that the increase in individuals intending to complete an AD may be attributed to the growing interest and knowledge regarding the “Life-Sustaining Treatment Decisions Act”, along with the progression of an aging society.

This study identified religious affiliation and knowledge of ADs as significant associated factors of caregivers’ intention to complete an AD. First, caregivers with religion were approximately 4.36 times more likely to intend to complete an AD compared with those without religion. This finding suggests that religious values may play a critical role in shaping attitudes toward EOL preparation and personal autonomy. Religious teachings often address the meaning of life and death, moral responsibility, and the limits of medical intervention—all of which may inform caregivers’ perspectives on ACP [33,34]. Spiritually sensitive education and counseling could be beneficial in supporting caregivers’ decision making. Awareness of religious beliefs and values around death and dying could be useful in preparing health professionals for ACP with caregivers and patients. Otherwise, according to a nationwide study conducted in the United States among participants aged 50 and older, those with a higher religious belief at EOL had lower odds of AD completion [35]. Religious influences on AD intentions may vary significantly to cultural norms. In future studies, multiple dimensions of religion, objectivity, and subjectivity, should be considered when studying health care preferences and decision making during EOL care.

Greater knowledge of ADs was associated with a higher likelihood of intending to complete one (OR = 1.16). This finding aligns with previous studies [21,36], suggesting that providing sufficient education and information about ADs to family caregivers is a crucial factor in enhancing their intention to complete one. This result emphasizes the importance of ensuring caregivers have access to accurate and practical information about ADs, including their purpose, legal procedures, and application. In this study, most items assessing basic concepts and general information about ADs had a correct response rate exceeding 80%. However, items addressing more specific aspects, including proxy designation, institutional procedures, documentation processes, and the scope of hospice and palliative care, scored below the average correct response rate of 60.7%. These findings are consistent with those of Yeom & Seo (2019) [31], who studied older adults, and Ryu (2022) [37], who examined the general public. This suggests the need to provide not only basic information about the advance healthcare directive system but also detailed and practical information regarding hospice and palliative care, along with the process of completing an AD. Therefore, a more structured educational program is needed to address this gap.

On the other hand, preferences for EOL care and attitudes toward withdrawing LST did not show a significant correlation with the intention to complete an AD. This may be due to a disconnect between personal treatment preferences and the legal or administrative action required to formalize those preferences through an AD [38]. Additionally, family caregivers of patients with dementia experience a high caregiving burden, which increases their risk of stress and depression and may lead to neglecting their own health management [34]. As a result, EOL planning for their own may become a lower priority for them. This may explain the weaker association between caregiving burden and the intention to complete an AD.

Although caregivers often play a critical role—especially when patients lose their decision-making capacity (e.g., in dementia)—they have not been the primary focus in most disease-related research [39]. Most studies have focused on the general public, health professionals, and students. Studies that specifically examine family caregivers’ knowledge of and intentions toward completing ADs are relatively scarce [10,40]. This study provides valuable insight into the factors associated with AD completion intentions among family caregivers of patients with dementia. By identifying religion and knowledge of ADs as key influencing factors, the findings highlight the need for targeted, educational strategies and policy support to promote informed EOL decision making. Future research should expand the sample size and explore the perspectives of patients and healthcare providers to develop more comprehensive interventions.

This study has several limitations. It used a cross-sectional design, which limits causal inference. This study was conducted through an online survey, which may have limitations in reaching certain age groups or individuals with low digital accessibility. Additionally, the sample size was not large enough to generalize the findings. Increasing the sample size or applying cross-validation techniques would improve reliability of the findings. This study was conducted within the specific cultural and healthcare context of Asia, and caution should be exercised when generalizing the findings to other countries or cultural settings. The reliability for the “pain management” subdomain of preference for EOL care was relatively low (Cronbach’s α = 0.41), likely due to the small number of items (three) in this subdomain. The reliability of the knowledge of ADs and attitudes toward withdrawing LST instruments was slightly lower than that reported in the original development studies. This discrepancy may be due to differences in participant characteristics (e.g., clinical nurses), such as educational and professional backgrounds, which could have influenced how respondents interpreted and responded to the items. Future research is recommended to further validate and refine these instruments across diverse populations and settings to improve their reliability.

## 5. Conclusions

These findings suggest that family caregivers’ religious backgrounds and knowledge of ADs may contribute to greater emphasis on their EOL preparedness and self-determination. To promote informed decision making, it is essential to implement targeted educational interventions—such as community-based workshops, telehealth counseling, and in-clinic guidance—that enhance caregivers’ understanding of ADs and empower them in their caregiving roles.

## Figures and Tables

**Table 1:** General characteristics of participants (n = 140).

Characteristics	Category	n (%)	M ± SD
Age (years)	20~29	10 (7.1)	41.50 ± 8.48
	30~39	46 (32.9)	
	40~49	62 (44.3)	
	≥50	22 (15.7)	
Gender	Male	20 (14.3)	
	Female	120 (85.7)	
Religion	No	79 (56.4)	
	Yes	61 (43.6)	
Education level	≤High school	36 (25.7)	
	≤Bachelor’s degree	104 (74.3)	
Income level	Low	19 (13.6)	
	Average	101 (72.1)	
	High	20 (14.3)	
Family cohabitation with patient	Living with	85 (60.7)	
	Living separately	55 (39.3)	
Other family members providing care	Yes	107 (76.4)	
	No	33 (23.6)	
Subjective health status	Healthy	80 (57.1)	
	Fair	53 (37.9)	
	Unhealthy	7 (5.0)	
Relationship to the patient	Spouse or child	103 (73.5)	
	Grandchildren or daughter-in-law	37 (26.4)	
Functional status of the patient	Minimal assistance	50 (35.7)	
	Moderate or greater assistance	90 (64.3)	
Type of dementia care provided	Institutional care	22 (15.7)	
	Day care center	57 (40.7)	
	Family-provided care	61 (43.6)	

M—Mean, SD—Standard Deviation.

**Table 2:** Knowledge of advance directives, preferences for EOL care, attitudes toward withdrawing life-sustaining treatment, and intention to complete an advance directive (n = 140).

Variables	n (%)	M ± SD	Range
Knowledge of ADs	-	12.65 ± 3.30	0~21
Preferences for EOL Care	-	3.12 ± 0.56	1~5
Pain management	-	3.96 ± 0.68	1~5
Role of family	-	3.64 ± 0.97	1~5
Spirituality	-	3.64 ± 0.97	1~5
Decision making by healthcare professionals	-	3.07 ± 1.13	1~5
Autonomous decision making	-	2.28 ± 0.83	1~5
Attitudes toward withdrawing LST	-	3.90 ± 0.50	1~5
Intention to complete an AD	-	-	-
Yes	119 (85.0)	-	-
No	21 (15.0)	-	-

M—mean, SD—standard deviation, ADs—advance directives, EOL—end-of-life, LST—life-sustaining treatment, AD—advance directive.

**Table 3:** Differences in the intention to complete an advance directive according to general characteristics (n = 140).

Characteristics	Category	Intention to Complete an AD		χ^2^ (*p*)
		Yes	No	
		n (%)	n (%)	
Age (years)	20~29	6 (60.0)	4 (40.0)	5.88 (0.117)
	30~39	40 (87.0)	6 (13.0)	
	40~49	55 (88.7)	7 (11.3)	
	≥50	18 (81.8)	4 (18.2)	
Gender	Male	14 (70.0)	6 (30.0)	(0.082)
	Female	105 (87.5)	15 (12.5)	
Religion	No	61 (77.2)	18 (22.8)	(0.004 *)
	Yes	58 (95.1)	3 (4.9)	
Education level	≤High school	31 (86.1)	5 (13.9)	(1.000)
	≤Bachelor’s degree	88 (84.6)	16 (15.4)	
Income level	Low	17 (89.5)	2 (10.5)	0.96 (0.620)
	Average	84 (83.2)	17 (16.8)	
	High	18 (90.0)	2 (10.0)	
Family cohabitation with patient	Living with	73 (85.9)	12 (14.1)	(0.810)
	Living separately	46 (83.6)	9 (16.4)	
Other family members providing care	Yes	92 (86.0)	15 (14.0)	(0.581)
	No	27 (81.8)	6 (18.2)	
Subjective health status	Healthy	69 (86.3)	11 (13.8)	1.96 (0.376)
	Fair	43 (81.1)	10 (18.9)	
	Unhealthy	7 (100.0)	0 (0.00)	
Relationship to the patient	Spouse or child	91 (88.3)	12 (11.7)	(0.104)
	Grandchild or daughter-in-law	28 (75.7)	9 (24.3)	
Functional status of the patient	Minimal assistance	37 (74.0)	13 (26.0)	(0.012 *)
	Moderate or greater assistance	82 (91.1)	8 (8.9)	
Type of dementia care provided	Institutional care	21 (95.5)	1 (4.5)	2.97 (0.226)
	Day care center	49 (86.0)	8 (14.0)	
	Family-provided care	49 (80.3)	45 (19.7)	

*: *p* < 0.05. AD—advance directive.

**Table 4:** Correlation between knowledge of advance directives, preferences for end-of-life care, attitudes toward withdrawing life-sustaining treatment, and intention to complete an advance directive.

Variables	Knowledge of ADs	Preferences for EOL Care	Attitudes Toward Withdrawing LST	Intention to Complete an AD
	r (*p*)			
Knowledge of ADs	1			
Preferences for EOL Care	−0.48 ** (0.000)	1		
Attitudes Toward Withdrawing LST	0.38 ** (0.000)	−0.15 (0.084)	1	
Intention to Complete an AD	0.23 * (0.007)	−0.08 (0.323)	0.11 (0.206)	1

*: *p* < 0.05, **: *p* < 0.001. ADs—advance directives, EOL—end-of-life, LST—life-sustaining treatment, AD—advance directive.

**Table 5:** Factors associated with the intention to complete an advance directive (n = 140).

Variables	B	SE	OR	Wald	95% CI	*p*
Constant	0.98	0.85	0.37	1.35		0.245
Religion *	1.47	0.67	4.36	4.82	1.171–16.265	0.028
Functional status of the patient **	0.88	0.53	2.41	2.78	0.856–6.803	0.096
Knowledge of ADs	0.15	0.07	1.16	4.55	1.012–1.332	0.033
		Nagelkerke R^2^ = 0.222, *p* < 0.05				

*: ref. no religion. **: ref. moderate or greater assistance. SE—standard error, OR—odds ratio, CI—confidence interval, ADs—advance directives.

## Data Availability

The original contributions presented in this study are included in the article. Further inquiries can be directed to the corresponding author.

## Abbreviations

The following abbreviations are used in this manuscript:

ADs	advance directives
EOL	end-of-life
LST	life-sustaining treatment

## Appendix A

**Table A1:** Knowledge of advance directives.

No	Questionnaire Items	Percentage of Correct Responses (%)
1	Hospice and palliative care provide medical services that support terminally ill patients in experiencing a natural and comfortable passing.	80. 7
2	If hospice and palliative care are provided, the administration of painkillers is discontinued.	58.6
3	When receiving hospice and palliative care, basic medical services such as nutritional support are provided.	77.9
4	Terminally ill patients lack decision-making capacity.	76.4
5	End-of-life patients lack decision-making capacity.	60.7
6	Life-sustaining treatment refers to the act of treating diseases.	81.4
7	Cardiopulmonary resuscitation (CPR), as part of life-sustaining treatment, is performed in cases of cardiac or respiratory arrest.	56.4
8	Any adult aged 19 or older may prepare an advance directive.	82.9
9	An advance directive is a document in which an adult specifies in advance whether they wish to receive or decline life-sustaining treatment in the event they lose decision-making capacity.	90.0
10	An advance directive can be completed by family members instead of the individual.	42.9
11	The person completing the advance directive can designate a proxy to make medical decisions on their behalf.	12.1
12	An advance directive must be completed at designated institutions only.	61.4
13	To complete an advance directive, professional assistance from a doctor or nurse is required.	33.6
14	An advance directive can be modified or revoked at any time.	82.1
15	A life-sustaining treatment plan is a document prepared by terminal patients nearing end-of-life, specifying whether they wish to receive or decline life-sustaining treatment.	86.4
16	A life-sustaining treatment plan must be prepared after the doctor directly explains it to the patient.	57.1
17	Once a life-sustaining treatment plan is prepared, it cannot be altered.	79.3
18	A life-sustaining treatment plan can be prepared based solely on the opinions of the patient’s family instead of the patient themselves.	41.4
19	A life-sustaining treatment plan is a document prepared by a doctor.	33.6
20	Instead of a life-sustaining treatment plan, another form such as a DNR (do not resuscitate order) may be used.	20.7
21	After completing a life-sustaining treatment plan, all medical care, including painkillers and antibiotics, is discontinued.	51.4
Total		60.7

## Appendix B

**Table A2:** Attitudes toward withdrawing life-sustaining treatment.

No	Questionnaire Items	Mean ± SD
1 *	Even for patients in an irreversible condition, all possible treatment methods should be utilized to extend their lives.	2.18 ± 1.23
2	If an irreversible patient and their family request the withdrawing life-sustaining treatment due to financial difficulties, it should be allowed.	4.19 ± 0.84
3	If an irreversible patient chooses to withdraw life-sustaining treatment over enduring the pain of treatment, it is a decision made for the patient’s benefit.	4.26 ± 0.83
4	For elderly patients in an irreversible condition, withdrawing life-sustaining treatment can be considered a way to conclude their remaining days.	4.16 ± 0.94
5	If an irreversible patient or their family requests the withdrawing life-sustaining treatment based on religious beliefs, it should be respected.	4.30 ± 0.83
6	If an irreversible patient and their family refuse intubation, even if it is deemed necessary, it should not be performed.	4.04 ± 0.95
7	Even if an irreversible patient’s blood pressure drops, if the patient or their family wishes, the administration of blood pressure-raising medication should be stopped.	4.04 ± 0.91
8 *	Cardiopulmonary resuscitation (CPR) should be performed if an irreversible patient experiences cardiac arrest.	2.70 ± 1.43
9	Objective and ethical guidelines are necessary when deciding to withdraw life-sustaining treatment.	4.03 ± 1.04
10	Families have the right to decide on a patient’s death.	4.17 ± 0.96
11	Patients have the right to decide on their own death.	4.39 ± 0.85
12	Withdrawing life-sustaining treatment for an irreversible patient should be permitted for organ donation purposes.	3.26 ± 1.16
13	If the patient’s family wishes, the mechanical ventilator for an unconscious irreversible patient should be stopped.	4.09 ± 0.96
14	Gradually reducing artificial respiration for an unconscious irreversible patient at the family’s request is a considerate approach for the patient.	4.21 ± 0.84
15	For patients without dependents in an irreversible condition, withdrawing life-sustaining treatment based on medical judgment is advisable.	4.17 ± 0.87
16	If the patient’s family requests voluntary discharge of an irreversible patient, it should be granted upon receiving signatures from immediate family members.	4.22 ± 0.91
17	For irreversible patients where cardiac arrest is anticipated, obtaining consent for a DNR (do not resuscitate order) is advisable.	4.26 ± 0.78
18 *	Even for irreversible patients admitted to the hospital, basic medication such as fluids and antibiotics should be provided.	3.83 ± 1.14
19 *	It is unacceptable for medical professionals to simply watch a patient die without providing any treatment or care.	2.89 ± 1.27

*: reverse-scored item.
